# Synchronous three-dimensional detection method for multiple parameters of wind fields based on vector principle

**DOI:** 10.3389/fpls.2022.1003659

**Published:** 2022-10-18

**Authors:** Shenghui Yang, Wenwei Li, Xingxing Liu, Zimeng Wang, Yongjun Zheng, Yu Tan, Han Feng

**Affiliations:** ^1^ College of Engineering, China Agricultural University, Beijing, China; ^2^ The Ministry of Education Engineering Research Center of Modern Agricultural Equipment and Facilities, Beijing, China

**Keywords:** wind, flow fields, vector, multidimensional systems, detection, sensors

## Abstract

In the area of air-assisted spray, conventional detection of speed and direction of the wind fields for spray are separately conducted, and multiple kinds of sensors have to be laid on each coordinate axis during multidimensional detection. It limits the optimization of operation effect of sprayers based on wind-field distribution characteristics. This paper proposes a novel detection method to achieve synchronous measurement of wind speed and direction in three dimensions. Wind flow was considered as vectors and the sensing structure with a regular triangular pyramid shape supported by cantilever pieces was established. Strain gauges were utilized to detect the deformation in each direction by the wind thrust onto a ball before and after wind flow. Moreover, the calculation models of wind speed and direction were developed respectively based on the relationship of ‘strains-force-wind pressure-wind velocity’ and the principle of space operation of vectors, so multiple parameters of wind fields could be obtained simultaneously. Calibration was conducted based on a wind tunnel and the Testo 405i anemometers. The results showed that: the minimum relative error of wind-speed values was about 0.06%, while the maximum was about 10%. The average relative error of all the directions was less than 5%. Furthermore, the measurement of the wind among artificial tree canopies demonstrated that the proposed method could effectively measure both speed value and direction of the wind among canopies, and it also helped to find the wind distribution characteristics of the fan, SFG4-2R. The results highlighted both the reliability and the practical meaning of the proposed method, which could be a technical solution for measuring and evaluating wind-field characteristics of sprayers.

## 1 Introduction

Air-assisted spray, including ground air-assisted spray and Unmanned Aerial Vehicles (UAVs) spray, has been widely used for plant protection. With the help of strong wind fields, droplets are delivered onto targets. At present, droplet deposition in crop canopies is not ideal during spraying, characterizing the issues of uneven distribution, inadequate penetration and significant drift (Pascuzzi et al, 2020; Zheng et al, 2020; Zhang et al, 2020). The fundamental reason is that the attenuation law of the wind field influenced by canopies has not been clearly studied, whose core difficulty is lacking effective detection approaches for the speed and flow direction of the wind in canopies. If there is a breakthrough in the real-time detection method of wind field changes in canopies, it will be of practical significance to help to improve the effect of air-assisted spray based on detection results.

Currently, the speed and direction of wind fields are separately measured by using different types of sensors, and most studies are just about speed quantification. In terms of the measurement of wind-speed values, impeller-type, thermosensitive-type and cup-type anemometers are commonly utilized. The majority of the research was to verify the consistency between Computational Fluid Dynamics (CFD) models and trials or to investigate wind-speed distribution in certain conditions. For impeller-type anemometers, Jiyu and Yubin et al. used them to investigate the downwash wind speed of a UAV, SUMA18 (Li et al, 2015a; Li et al, 2019b), as well as the relationship between wind fields and the distribution of pollen (Li et al, 2017c) or droplets (Chen et al, 2017). The new research of this team was continuous to study the consistency between the distribution of UAV wind fields and that of droplet depositions (Lan et al, 2021; Zhan et al, 2022). All these studies mainly focused on the same type of UAV. Yang et al. (2017) conducted trials to verify the accuracy of downwash CFD models by the Kestrel 4500 anemometer, and Zhang et al. (2019a) and Guo et al. (2020) also did similar works by using Kestrel 4500 or GM8902+. For thermosensitive-type anemometers, Yang et al. (2022a) utilized Testo 405i to examine the transmission of UAV downwash in corn canopies, and Zhang et al. (2019b) also applied this anemometer to observe the conformity between downwash CFD models and the test results. In addition, Wang et al. (2021) developed a kind of thermosensitive anemometer to measure the wind field of a six-rotor UAV, while Cheng et al. (2021) used CTA-type anemometer to measure that of an unmanned helicopter. Cup-type anemometers are generally taken for agricultural meteorological measurement in outdoor conditions (Xing et al, 2015), not for air-assisted spray. Although the wind-speed sensors mentioned above in these studies have a fast response and good stability, they can only detect single-dimensional values. If three-dimensional results are required, such sensors have to be separately laid on each axis. Meanwhile, each sensor takes up space and may be interfered by each other so that the wind field shape may be affected, which will lead to inaccurate results.

In terms of wind direction measurement, wind vane sensors are conventional devices, often adopted for acquiring meteorological and environmental parameters rather than for air-assisted spray. For instance, Li et al. (2021) used wind vanes to measure the wake variation of horizontal axis wind turbine. Sharma et al. (2018) used them to estimate the accuracy of the wind resource of a site. However, wind vanes can only measure an angle from 0 to 360° in a horizontal plane, not suitable for a vertical layout. Therefore, the three-dimensional measurement of wind direction needs another approach.

In order to achieve the synchronous detection of both wind speed and wind direction, several techniques have been developed, typically ultrasonic anemometers and Micro-Electro-Mechanical System (MEMS) anemometers. Ultrasonic anemometers measure by using the frequency difference between the transmitter and the receiver, while MEMS-type ones mainly rely on the change of pressure or thermal fields. Researchers have used ultrasonic ones to measure the downwash of a UAV (Tang et al, 2019) and environmental parameters (Schramm et al, 2019) to control drifts, whilst the MEMS type is generally exploited in non-agricultural areas (Jiang, 2021). Irrespective of the ultrasonic and MEMS types, the measurement results are two-dimensional. Even though a study was related to a three-dimensional wind-field measurement device to observe the wind field of a hovering UAV (Wu et al, 2019), it described the distribution of wind-field intensity rather than wind speed, let alone wind direction. Hence, there is a shortage of the three-dimensional detection method of wind speed and direction.

In fact, if a particular substance in a specific space can be called a “field” in physics, it must consist of vectors in mathematics, and so is the wind field for air-assisted spray. Any vector in a wind field, $\vec{v}$ , is composed of three basis vectors ( ${\vec{v}}_{x}$ , ${\vec{v}}_{y}$ and ${\vec{v}}_{z}$ ) on the *x*, *y* and *z* axes. If the length (size) and direction of each base vector can be measured, a unique wind-speed vector will be determined, and the angle between the vector and each coordinate axis will also be unique.

This paper proposes a synchronous three-dimensional detection method for multiple parameters of wind fields based on the vector principle. A novel sensing structure and its matching calculation models were developed to tackle the practical difficulty of wind-field detection in the area of air-assisted spray.

## 2 Materials and methods

### 2.1 Materials

#### 2.1.1 Hardware

**Figure 1:**
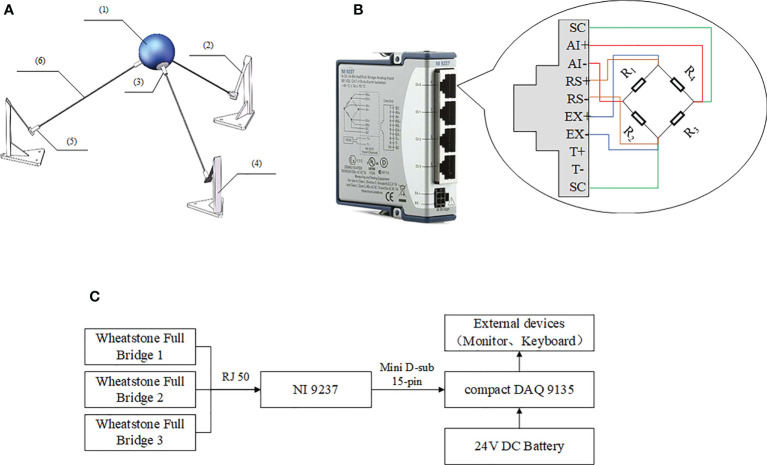
The design of the measurement scheme, where **(A)** shows the shape and materials, **(B)** shows the connection between each Wheatstone Full Bridge and NI 9237, and **(C)** shows the combination and connections of each component. (1) a smooth hollow plastic ball, (2) cantilever pieces, (3) ball seats, (4) bases, (5) jacks and (6) carbon fiber tubes.

**Figure 1** shows the designed system of measurement. The materials contained a smooth hollow plastic ball, three cantilever pieces, three bases, three ball seats and three carbon fiber tubes. The system was a regular triangular pyramid, and the fiber tubes were mutually perpendicular. The radius of the ball, *R*, was 40 mm, and its mass was only about 40 g. Moreover, Wheatstone Full Bridge was utilized on each cantilever piece to detect and measure strain variations. The strain gauges were resistive type, BFH350-6AA, and NI 9237 combined with NI Compact DAQ 9135 was employed to achieve strain data acquisition and saving. The key specifications of BFH350-6AA, NI 9237 and cantilever pieces are listed in **Table 1**.

**Table 1:** The key specifications of BFH350-6AA and NI 9237.

Materials/Device	Parameters	Values with unit	Unit/Remarks
BFH350-6AA	Resistance	350 ± 0.1	Ω
	Base length × base width	10.3×3.9	mm
	Grid length × grid width	6×2.9	mm
	Sensitivity	2.0 ± 1%	—
	Tolerance to nominal values	1000 ± 3	Ω
	Tolerance to means	≤0.5	Ω
NI 9237	Excitation	3.3	V/Four 350 Ω Full Bridges
	Sampling rate	$f_{s}=\frac{f_{M}}{256\cdot n}$	*f_M_* is the principal time base, and n is an integer from 1 to 31
	Accuracy	± 100	ppm·max.value^-1^
	Excitation noise	100	μV_rms_
	Conversion accuracy	2.9802	nV·V^-1^·LSB^-1^
Cantilever piece	Material	65 Mn	—
	elastic modulus	1.97×10^11^	Pa
	Width of strain area	7.00	mm
	Thickness	0.50	mm
	Max.length of strain area	55.00	mm

When flowing around a circular sphere, fluid will form a thrust on it in the flow direction (Liu et al, 2017). Therefore, if a force change in this direction can be measured, the thrust will be obtained. Then, the wind speed value can be calculated according to the relations between force, wind pressure and wind speed.

#### 2.1.2 Software

**Figure 2:**
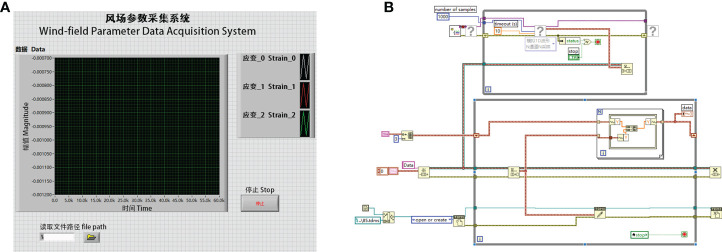
The developed software, where **(A)** is the interface of the software and **(B)** is the LABVIEW code of it.

The data acquisition software was developed using LABVIEW 2016 (**Figure 2**), including real-time data collection, saving and viewing. Three data channels from three Full Bridge could be acquired separately and saved into the same document.

### 2.2 Measurement method of wind vector

#### 2.2.1 Measurement of wind vector length

**Figure 3:**
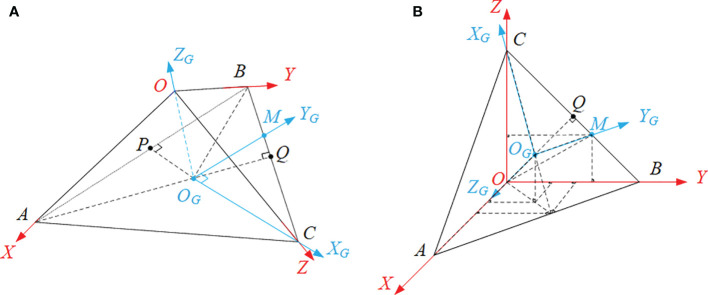
The coordinate systems, including the Sensing Coordinate System (marked by red), *O*-*XYZ*, and the Ground Coordinate System (marked by blue), *O_G_*-*X_G_Y_G_Z_G_*, where **(A)** is the standard view of the coordinate systems whilst **(B)** is the oblique axonometric drawing for the convenience of calculation.

As shown in **Figure 3**, the ball center was taken as the origin, *O*, to establish the Sensing Coordinate System (SCS, marked by red), *O*-*XYZ*, while the center of Triangle *ABC* was taken as the origin, *O_G_*, to establish the Ground Coordinate System (GCS, marked by blue), *O_G_*-*X_G_Y_G_Z_G_*.

**Figure 4:**
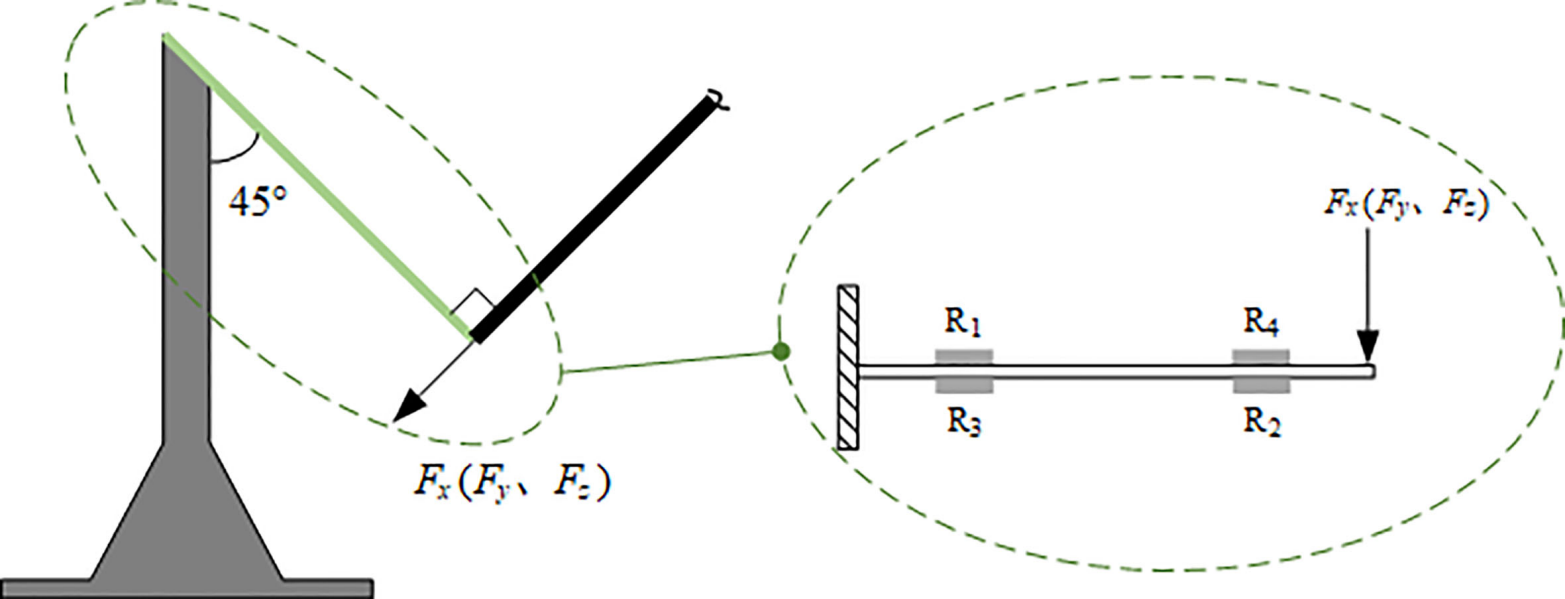
The force analysis of the cantilever pieces of the three directions of the Sensing Coordinate System.

In the SCS, the force analysis for the cantilever piece of any direction can be equivalent to a cantilever beam model (**Figure 4**). Since the solution process of the force in each direction was the same, that in the *X* direction was taken as an instance for illustration.

In terms of the cantilever pieces, if the elastic modulus is *E*, the strain without flow around is *ϵ*_*x*_0__, the bending section coefficient is *W*, and the distance between the two strain gauges on the same side is *l*, the initial component of the force generated by the gravity of both the ball and the fiber tubes in the *X* direction, *F*_*x*_0__, should be


(1) $$F_{x_{0}}=\frac{\epsilon_{x_{0}}\cdot E\cdot W}{l}$$

When wind flows around the ball, the strain will be changed to *ϵ*_*x*_1__, so the component of the force in this direction, *F*_*x*_1__, will be


(2) $$F_{x_{1}}=\frac{\epsilon_{x_{1}}\cdot E\cdot W}{l}$$

The force difference between these two conditions,

*F*_*p*_*x*__, is


(3) $$F_{p_{x}}=F_{x_{1}}-F_{x_{0}}=\frac{EW}{l}(\epsilon_{x_{1}}-\epsilon_{x_{0}})$$

In addition, the bending section coefficient, *W*, can be calculated by


(4) $$W=\frac{bh^{2}}{6}$$

, where *b* is the width of the cantilever pieces and *h* is its thickness.

Thus, the force difference (which is also called the wind thrust),

*F*_*p*_*x*__, should be


(5) $$F_{p_{x}}=\frac{Ebh}{6l}(\epsilon_{x_{1}}-\epsilon_{x_{0}})$$

According to Equation (5), the magnitude and direction of the thrust can be calculated by the strain variations.

Moreover, the relationship between force and wind pressure is


(6) $$p_{x}=\frac{F_{p_{x}}}{A}$$

, where A is the flow area, equal to the surface area of the ball in this study: A=4π*R* ^2^.

The relation between the wind pressure and wind speed is (Liu et al, 2021):


(7) $$p_{x}=\frac{v_{x}2^{}}{1600}\times 10^{3}$$

Hence, based on the Equations from (5) to (7), if $\vec{i}=(1,0,0)$ is the unit base vector of the *X* direction in the SCS, the wind vector of this direction, $\vec{v_{x}}$ , should be

$$\begin{array}{l} \vec{v_{x}}=\sqrt{\frac{200Ebh^{2}\left|\epsilon_{x_{1}}-\epsilon_{x_{0}}\right|}{3\pi lR^{2}}\times 10^{-3}}\cdot \frac{\epsilon_{x_{1}}-\epsilon_{x_{0}}}{\left|\epsilon_{x_{1}}-\epsilon_{x_{0}}\right|}\cdot \vec{i}\text{ , or }\\ \vec{v_{x}}=\left\{ \begin{array}{l} \left(\sqrt{\frac{200Ebh^{2}(\epsilon_{x_{1}}-\epsilon_{x_{0}})}{3\pi lR^{2}}\times 10^{-3}},0,0\right),\epsilon_{x_{1}}>\epsilon_{x_{0}}\\ \left(-\sqrt{\frac{200Ebh^{2}(\epsilon_{x_{0}}-\epsilon_{x_{1}})}{3\pi lR^{2}}\times 10^{-3}},0,0\right)\text{, }\epsilon_{x_{1}}<\epsilon_{x_{0}} \end{array}\right. \end{array}$$

For the same reason, that of the *Y* and *Z* directions in the SCS should be

$$\begin{array}{l} \vec{v_{y}}=\sqrt{\frac{200Ebh^{2}\left|\epsilon_{y_{1}}-\epsilon_{y_{0}}\right|}{3\pi lR^{2}}\times 10^{-3}}\cdot \frac{\epsilon_{y_{1}}-\epsilon_{y_{0}}}{\left|\epsilon_{y_{1}}-\epsilon_{y_{0}}\right|}\cdot \vec{j}\text{, or}\\ \vec{v_{y}}=\left\{ \begin{array}{l} \left(0,\sqrt{\frac{200Ebh^{2}\left(\epsilon_{y_{1}}-\epsilon_{y_{0}}\right)}{3\pi lR^{2}}\times 10^{-3}},0\right),\epsilon_{y_{1}}>\epsilon_{y_{0}}\\ \left(0,-\sqrt{\frac{200Ebh^{2}\left(\epsilon_{y_{0}}-\epsilon_{y_{1}}\right)}{3\pi lR^{2}}\times 10^{-3}},0\right)\text{, }\epsilon_{y_{1}}<\epsilon_{y_{0}} \end{array}\right. \end{array}$$

(9)


(10) $$\begin{array}{l} \vec{v_{z}}=\sqrt{\frac{200Ebh^{2}\left|\epsilon_{z_{1}}-\epsilon_{z_{0}}\right|}{3\pi lR^{2}}\times 10^{-3}}\cdot \frac{\epsilon_{z_{1}}-\epsilon_{z_{0}}}{\left|\epsilon_{z_{1}}-\epsilon_{z_{0}}\right|}\cdot \vec{k},\text{ or}\\ \vec{v_{z}}=\left\{ \begin{array}{l} \left(0,0,\sqrt{\frac{200Ebh^{2}\left(\epsilon_{z_{1}}-\epsilon_{z_{0}}\right)}{3\pi lR^{2}}\times 10^{-3}}\right),\epsilon_{z_{1}}>\epsilon_{z_{0}}\\ \left(0,0,-\sqrt{\frac{200Ebh^{2}\left(\epsilon_{z_{0}}-\epsilon_{z_{1}}\right)}{3\pi lR^{2}}\times 10^{-3}}\right)\text{, }\epsilon_{z_{1}}<\epsilon_{z_{0}} \end{array}\right. \end{array}$$

Finally, based on the Equations from (8) to (10), the three-directional wind vector and its length can be calculated by Equation (11) and Equation (12):


(11) $$\vec{v}=\vec{v_{x}}+\vec{v_{y}}+\vec{v_{z}}$$

(12) $$\vec{\left|v\right|}=\sqrt{\frac{200Ebh^{2}}{3\pi lR^{2}}\times 10^{-3}\times (\left|\epsilon_{x_{1}}-\epsilon_{x_{0}}\right|+\left|\epsilon_{y_{1}}-\epsilon_{y_{0}}\right|+\left|\epsilon_{z_{1}}-\epsilon_{z_{0}}\right|)}$$

Equation (12) shows that the measurement result of wind speed is only related to the strains before and after flow when the components of the device are fixed, which means the external conditions have less influence on wind speed measurement.

#### 2.2.2 Measurement of wind vector direction

If the angle between any wind vector and the unit base vectors of the three axes of the GCS can be calculated, the direction of this wind vector will be determined. Therefore, each point in **Figure 3** was firstly coordinated in the same coordinate system, the SCS, and then the principle of vector coordinate operation was applied to obtain the results.

If the edge length of the pyramid is *κ*, the coordinate of Point *A*, Point *B* and Point *C* will be (*κ*,0,0), (0, *κ*,0) and (0,0, *κ*), respectively. According to geometric relations, the coordinate of Point *M* and Point *O_G_* should be $\left(0,\frac{2\kappa }{3},\frac{\kappa }{3}\right)$ and $\left(\frac{\kappa }{3},\frac{\kappa }{3},\frac{\kappa }{3}\right)$ , respectively. Hence, the vectors $\vec{O_{G}C}$ , $\vec{O_{G}M}$ and $\vec{O_{G}O}$ can be solved and then unitized to be the unit base vectors of the GCS, $\vec{x_{G}}$ , $\vec{y_{G}}$ and $\vec{z_{G}}$ . Equation (13) are these unit base vectors:


(13) $$\left\{ \begin{array}{l} \vec{x_{G}}=\frac{\vec{O_{G}C}}{\left|\vec{O_{G}C}\right|}=\left(-\frac{\sqrt{6}}{6},-\frac{\sqrt{6}}{6},\frac{\sqrt{6}}{3}\right)\\ \vec{y_{G}}=\frac{\vec{O_{G}M}}{\left|\vec{O_{G}M}\right|}=\left(-\frac{\sqrt{2}}{2},\frac{\sqrt{2}}{2},0\right)\\ \vec{z_{G}}=\frac{\vec{O_{G}O}}{\left|\vec{O_{G}O}\right|}=\left(-\frac{\sqrt{3}}{3},-\frac{\sqrt{3}}{3},-\frac{\sqrt{3}}{3}\right) \end{array}\right.$$

Finally, based on the Equations from (8) to (13), the direction of the wind vector can be calculated as follows:


(14) $$\left\{ \begin{array}{l} \cos\alpha =\frac{\vec{x_{G}}\cdot \vec{v}}{\left|\vec{x_{G}}\right|\left|\vec{v}\right|}=-\frac{\sqrt{6}}{6}\cdot \frac{\sqrt{\left|\epsilon_{x_{1}}-\epsilon_{x_{0}}\right|}\cdot \frac{\epsilon_{x_{1}}-\epsilon_{x_{0}}}{\left|\epsilon_{x_{1}}-\epsilon_{x_{0}}\right|}+\sqrt{\left|\epsilon_{y_{1}}-\epsilon_{y_{0}}\right|}\cdot \frac{\epsilon_{y_{1}}-\epsilon_{y_{0}}}{\left|\epsilon_{y_{1}}-\epsilon_{y_{0}}\right|}-2\sqrt{\left|\epsilon_{z_{1}}-\epsilon_{z_{0}}\right|}\cdot \frac{\epsilon_{z_{1}}-\epsilon_{z_{0}}}{\left|\epsilon_{z_{1}}-\epsilon_{z_{0}}\right|}}{\sqrt{\left|\epsilon_{x_{1}}-\epsilon_{x_{0}}\right|+\left|\epsilon_{y_{1}}-\epsilon_{y_{0}}\right|+\left|\epsilon_{z_{1}}-\epsilon_{z_{0}}\right|}}\\ \cos\beta =\frac{\vec{y_{G}}\cdot \vec{v}}{\left|\vec{y_{G}}\right|\left|\vec{v}\right|}=-\frac{\sqrt{2}}{2}\cdot \frac{\sqrt{\left|\epsilon_{x_{1}}-\epsilon_{x_{0}}\right|}\cdot \frac{\epsilon_{x_{1}}-\epsilon_{x_{0}}}{\left|\epsilon_{x_{1}}-\epsilon_{x_{0}}\right|}-\sqrt{\left|\epsilon_{y_{1}}-\epsilon_{y_{0}}\right|}\cdot \frac{\epsilon_{y_{1}}-\epsilon_{y_{0}}}{\left|\epsilon_{y_{1}}-\epsilon_{y_{0}}\right|}}{\sqrt{\left|\epsilon_{x_{1}}-\epsilon_{x_{0}}\right|+\left|\epsilon_{y_{1}}-\epsilon_{y_{0}}\right|+\left|\epsilon_{z_{1}}-\epsilon_{z_{0}}\right|}}\\ \cos\varphi =\frac{\vec{z_{G}}\cdot \vec{v}}{\left|\vec{z_{G}}\right|\left|\vec{v}\right|}=-\frac{\sqrt{3}}{3}\cdot \frac{\sqrt{\left|\epsilon_{x_{1}}-\epsilon_{x_{0}}\right|}\cdot \frac{\epsilon_{x_{1}}-\epsilon_{x_{0}}}{\left|\epsilon_{x_{1}}-\epsilon_{x_{0}}\right|}+\sqrt{\left|\epsilon_{y_{1}}-\epsilon_{y_{0}}\right|}\cdot \frac{\epsilon_{y_{1}}-\epsilon_{y_{0}}}{\left|\epsilon_{y_{1}}-\epsilon_{y_{0}}\right|}+\sqrt{\left|\epsilon_{z_{1}}-\epsilon_{z_{0}}\right|}\cdot \frac{\epsilon_{z_{1}}-\epsilon_{z_{0}}}{\left|\epsilon_{z_{1}}-\epsilon_{z_{0}}\right|}}{\sqrt{\left|\epsilon_{x_{1}}-\epsilon_{x_{0}}\right|+\left|\epsilon_{y_{1}}-\epsilon_{y_{0}}\right|+\left|\epsilon_{z_{1}}-\epsilon_{z_{0}}\right|}} \end{array}\right.$$

, where *α*, *β* and *φ* are the angles between the wind vector and the *X_G_*, *Y_G_* and *Z_G_* axes of the Ground Coordinate System, respectively.

According to Equation (14), in terms of this determined design, the direction of wind vectors is associated with neither the material characteristics of the cantilever pieces nor the geometric features of the devices, only related to the strains before and after wind flow. Thus, the external influence of direction measurement has been kept to a minimum in theory to reduce errors.

### 2.3 Experiment schemes

#### 2.3.1 Calibration

**Figure 5:**
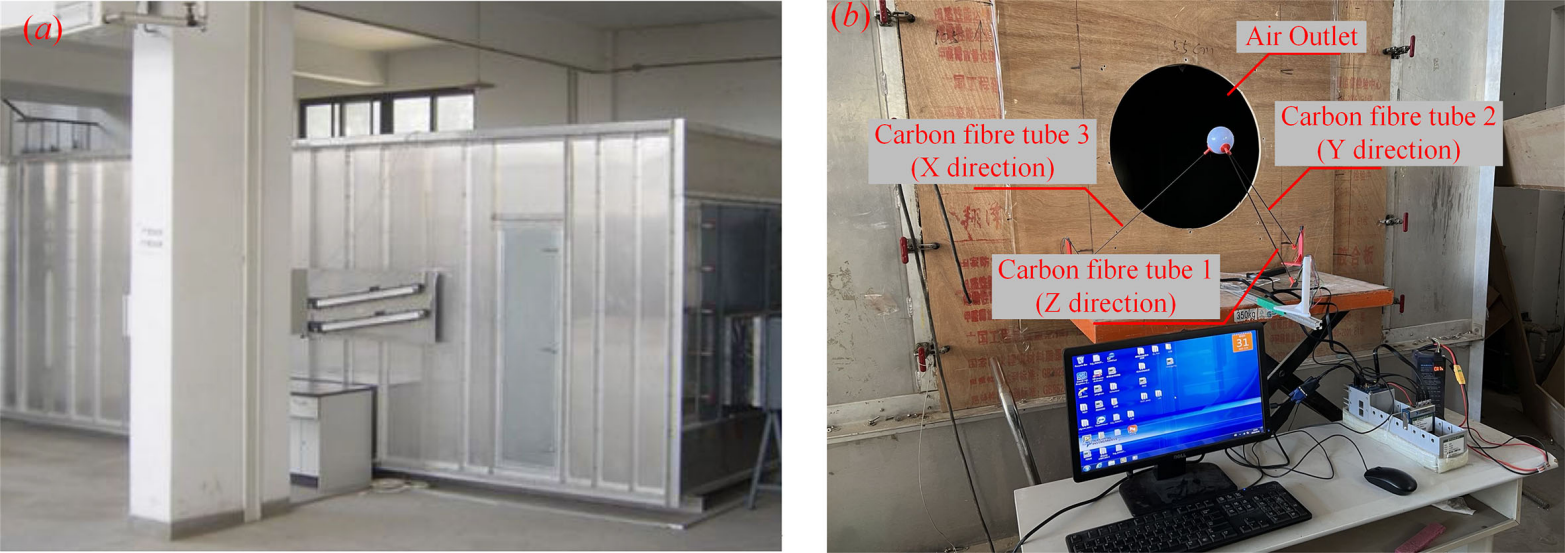
The utilized equipment and settings of the calibration tests, where **(A)** shows the used wind tunnel and **(B)** shows the layout of the tests.

**Figure 5A** shows the wind tunnel used for calibration, which is located on the East Campus of China Agricultural University. The air volume is adjustable at most 60000 m^3^·h^-1^, and the diameter of the air outlet is 555 mm. As shown in **Figure 5B**, the measurement device was placed about 0.5 m in front of the center of the air outlet to ensure full flow.

Eight-level air volumes were applied, including 1869 m^3^·h^-1^, 2386 m^3^·h^-1^, 3534 m^3^·h^-1^, 4260 m^3^·h^-1^, 5191 m^3^·h^-1^, 6134 m^3^·h^-1^, 7448 m^3^·h^-1^ and 8399 m^3^·h^-1^. The data were collected for more than 1 minute under each air volume condition. The maximum speed from the wind tunnel was approximately 14 m·s^-1^, while that from ground air-assisted sprayers and Unmanned Aerial Vehicles is generally less than about 12 m·s^-1^ (Yang et al, 2020b). Hence, the range of calibration was sufficient.

**Figure 6:**
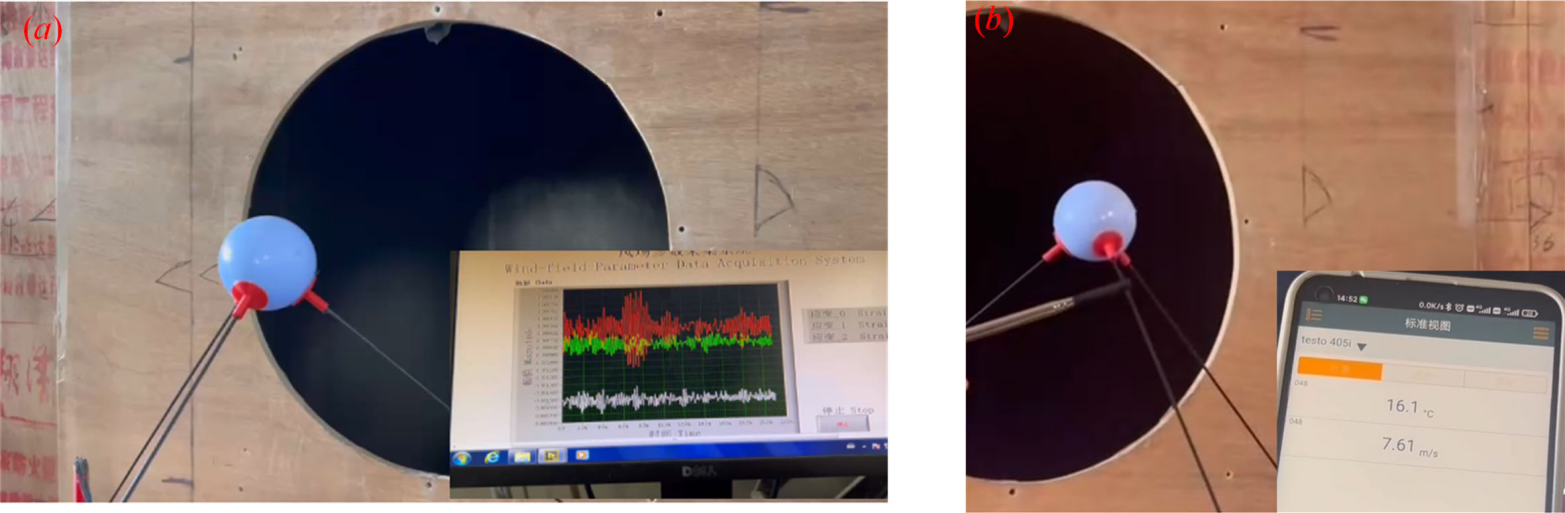
The process of the tests, where **(A)** shows the process of the measurement system and **(B)** shows that of the thermal anemometer.

**Figure 6** shows the process of the tests. Firstly, the measurement device collected the strains of three dimensions. Then, a thermal anemometer, Testo 405i by Testo Germany (**Table 2**), was used to measure the wind speed along the three fiber tubes, respectively. Finally, the wind speed data were calculated, and the calibration equations by the regression between measurement system results and anemometer ones were established.

**Table 2:** The key specifications of the thermal anemometer, Testo 405i.

Parameters	Values	Remarks
Measurement Range	0-30 m·s^-1^	
Sampling Frequency	0.5 Hz	
Resolution	0.01 m·s^-1^	
Accuracy	±(0.1 m·s^-1^ +5% Measured Value)	0-2 m·s^-1^
	±(0.3 m·s^-1^ +5% Measured Value)	2-15 m·s^-1^

After that, the eight-level air volumes were applied again to examine the calibration effect. The regression between the calibrated system results and the anemometer ones was conducted once more to analyze relevance. Meanwhile, the relative errors of the three-directional measurement were indicated, respectively.

#### 2.3.2 Measurement of the wind among tree canopies

**Figure 7:**
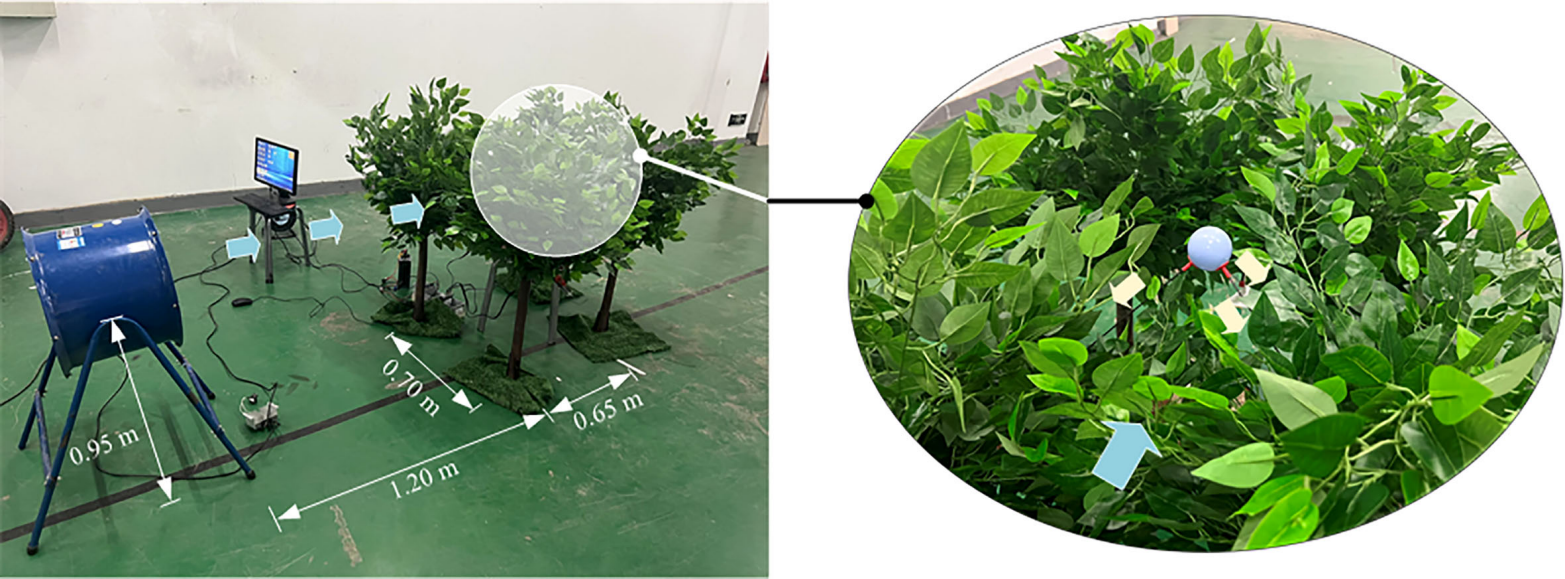
The setting of measurement of the wind among canopies.

After calibration, the system was exploited to measure the wind among tree canopies. The wind was from an axial-flow fan, SFG4-2R, which is commonly used on small Chinese air-assisted ground sprayers. As shown in **Figure 7**, the ball was placed among the artificial trees, and the distance from the fan to the tree was about 1.20 m. The process was: ①starting the measurement system, ② turning on the fan to reach the rated speed (2800 r·min^-1^), and ③switching off the fan. Real feature of the wind in canopies was investigated, and the data was collected for about 10 seconds.

### 2.4 Data processing methods

#### 2.4.1 Data processing for calibration

In stable wind condition, the measured strains and wind speeds significantly fluctuated around a mean. Therefore, Global Average Method (GAM) was applied to process the data from both the wind measurement system and the anemometer, as shown in Equation (15):


(15) $$\bar{v}=\frac{\sum_{i=1}^{n}v_{i}}{n}$$

Furthermore, the regression and relative errors Equation (16) were calculated using ORIGIN 2018. The data from the anemometer were taken as the standard to make comparisons of accuracy:


(16) $$e=\frac{\left|e_{ane}-e_{sys}\right|}{e_{ane}}$$

, where *e* is the relative error, *e*_*ane*_ is the data from the anemometer and *e*_*sys*_ is that from the measurement system.

#### 2.4.2 Data processing for in-canopy wind measurement

Due to wind variation caused by canopies, the measured strains were not fluctuated around a global mean. However, in a certain small period, strains still fluctuated around a local mean. Hence, Local Average Method (LAM) was utilized to process the data.

If the length of the data was *U* and the grouping interval was *u*, the local mean was calculated by Equation (17):


(17) $$v_{k+i}=\frac{\sum_{i=1}^{u}x_{i+ku}}{u}$$

, where *k*= $0,1,\ldots ,\frac{U}{u}-1$ . Then, the vector components in the GCS were calculated by Equation (18):


(18) $$\left\{ \begin{array}{l} \vec{v_{x_{G}}}=\vec{v}\cdot \cos\alpha \\ \vec{v_{y_{G}}}=\vec{v}\cdot \cos\beta \\ \vec{v_{z_{G}}}=\vec{v}\cdot \cos\varphi \end{array}\right.$$

Finally, the wind vectors were drawn by MATLAB 2019b based on their both starting points and GCS components.

## 3 Results and discussions

### 3.1 Calibration results

**Figure 8:**
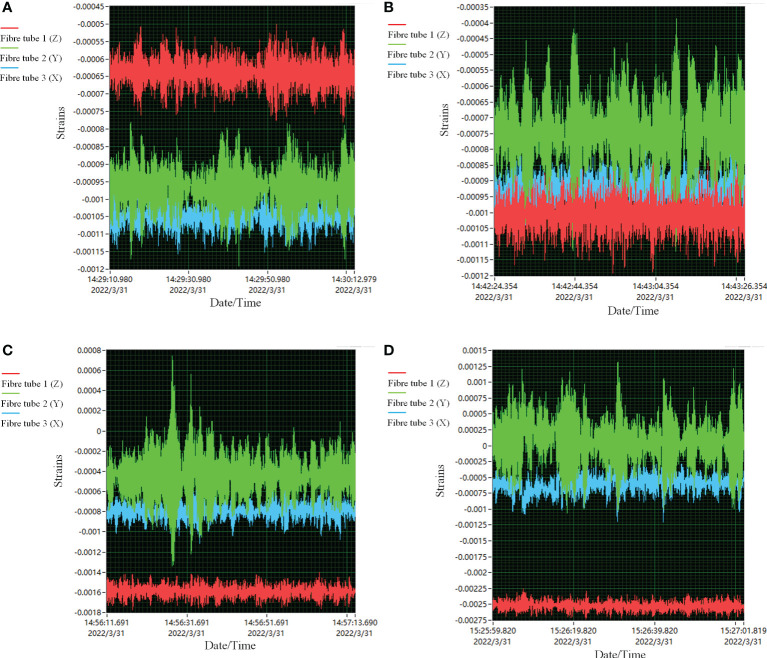
Some of the strain data collected by the measurement system, where the air volumes were 2386 m^3^·h^-1^, 4260 m^3^·h^-1^, 6134 m^3^·h^-1^ and 8399 m^3^·h^-1^ from **(A–D)**, respectively.

**Figure 8**, **9** are the collected data from the measurement system and the anemometer, respectively.

**Figure 9:**
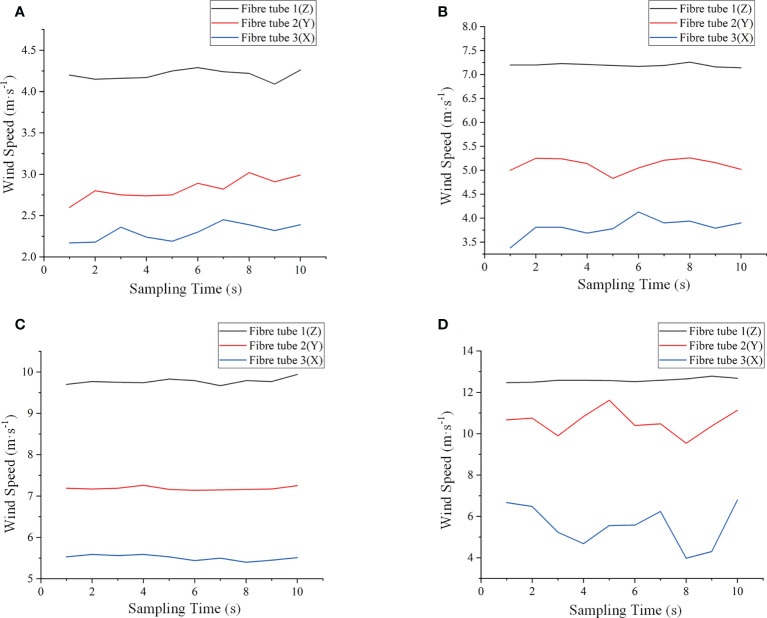
Some of the wind speed data from the thermal anemometer, where the air volumes were 2386 m^3^·h^-1^, 4260 m^3^·h^-1^, 6134 m^3^·h^-1^ and 8399 m^3^·h^-1^ from **(A–D)**, respectively.

**Figure 10:**
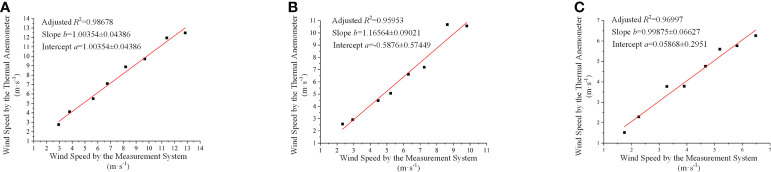
The regressions between the thermal anemometer and the measurement system, where **(A–C)** correspond to Carbon Fibre Tube 1, Carbon Fibre Tube 2 and Carbon Fibre Tube 3, respectively.

**Figure 10** shows the regression for calibration. The adjusted *R* ^2^ was 0.98678, 0.95953 and 0.96997, respectively, indicating a significant relevance of the results between the thermal anemometer and the measurement system.

According to **Figure 10**, the calibration equations for each direction of the Sensing Coordinate System were:


(19) $$\left\{ \begin{array}{l} Z=1.00354z+0.12686\\ Y=1.16564y-0.5876\\ X=0.99875x+0.05868 \end{array}\right.$$

, where *z*, *y* and *x* are the data from Tube 1, Tube 2 and Tube 3, respectively, while *Z*, *Y* and *X* are that from the thermal anemometer.

**Table 3** lists the result comparison between the anemometer and the calibrated measurement system using Equation (19). Three decimal places were kept based on the accuracy of the anemometer.

**Table 3:** The wind speed by the thermal anemometer and the calibrated measurement system.

Air Volume (m^3^·h^-1^)	Wind Speed by the Proposed Measurement System (m·s^-1^)			Wind Speed by the Thermal Anemometer (m·s^-1^)			Relative Error(%)		
	Fibre Tube 1(Z)	Fibre Tube 2(Y)	Fibre Tube 3(X)	Fibre Tube 1(Z)	Fibre Tube 2(Y)	Fibre Tube 3(X)	Fibre Tube 1(Z)	Fibre Tube 2(Y)	Fibre Tube 3(X)
1869	3.075	-1.970	-1.712	2.828	2.150	1.782	8.751	8.370	3.901
2386	3.865	-2.827	-2.181	4.097	2.748	2.285	5.655	2.886	4.547
3534	5.820	-4.532	-3.381	5.963	4.528	3.153	2.405	0.084	7.235
4260	6.852	-5.535	-3.743	6.493	5.502	4.169	5.527	0.606	10.211
5191	8.286	-6.702	-4.720	8.479	6.671	4.826	2.279	0.458	2.203
6134	9.804	-7.789	-5.148	9.775	7.774	5.495	0.298	0.193	6.319
7448	11.419	-9.929	-5.154	11.649	10.324	5.256	1.978	3.829	1.933
8399	12.938	-10.817	-6.416	12.838	10.616	6.412	0.776	1.893	0.062
Average Relative Error (%)							3.458	2.290	4.551

**Figure 11:**
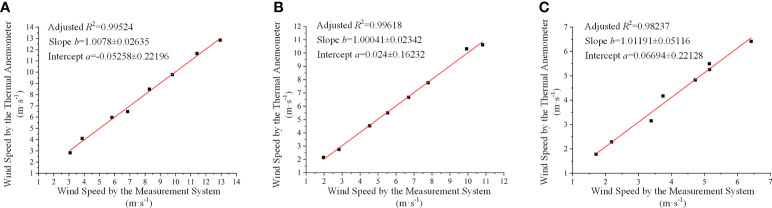
The regression between the anemometer and the calibrated measurement results, where **(A–C)** correspond to Carbon Fibre Tube 1, Carbon Fibre Tube 2 and Carbon Fibre Tube 3, respectively.

**Figure 11** is the regression between the anemometer and the calibrated measurement results.

According to **Table 3** and **Figure 11**, it can be illustrated that:

(1) the relevance after calibration was greatly improved. The adjusted *R* ^2^ was up to 0.99524, 0.99618 and 0.98237 (**Figure 11**), respectively. Meanwhile, all the slope values were about 1 and the intercepts were lower than 0.07. The calibration was proper.

(2) the proposed synchronous detection method and system could accurately and effectively measure wind speed, as the minimum relative error was about 0.06%, while the maximum error was about 10% (**Table 3**). The average relative error of all the directions of the SCS was less than 5%. Although the relative errors seemed to vary greatly, the maximum difference was only 1.4 m·s^-1^ in terms of the top wind speed of 14 m·s^-1^. In other words, the absolute error was still small enough.

(3) compared with the results from the anemometer, the proposed method and system could obtain the direction of wind flow since the calculated positive or negative values obviously indicated it (**Table 3**). Thus, it is achieved to acquire wind speed and wind direction synchronously.

### 3.2 Measurement of the wind among tree canopies

**Figure 12:**
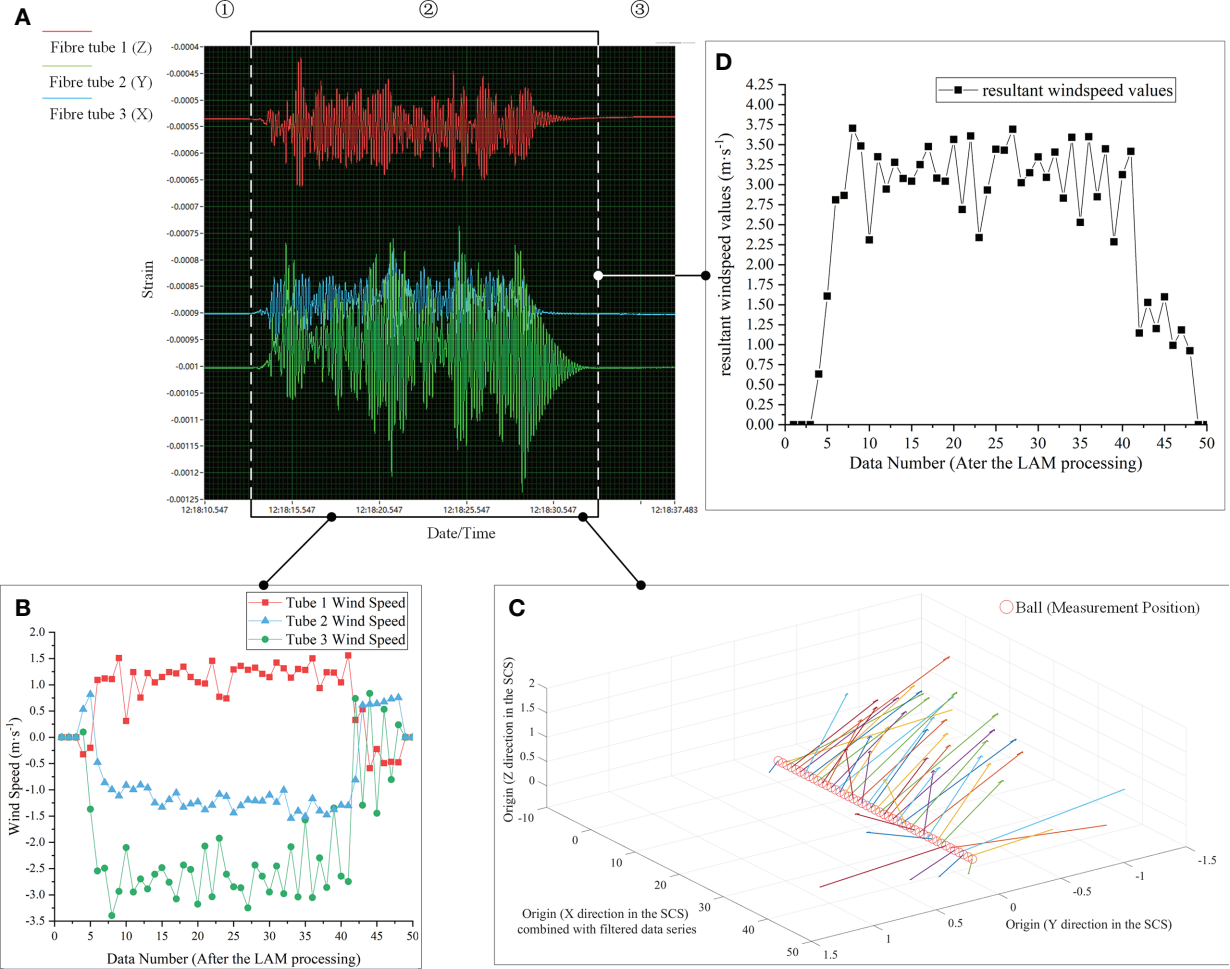
The result of measurement of the wind among tree canopies, where **(A)** is the strains measured by the system, **(B)** shows the wind speed of each SCS direction after the LAM processing, **(C)** illustrates the wind vector variations based on the LAM-processed data series, and **(D)** gives the resultant wind speed values based on each direction results shown in **(B)**.

**Figure 12** demonstrates the results of measurement of in-canopy wind, indicating that:

(1) The system could clearly present the three stages of the trial (①, ② and ③ in **Figure 12A**) and the variation of wind. In addition, one signal cycle was about 160 ms, so the grouping interval in Equation (17), *u*, was set as 160 for the LAM processing.

(2) The system could effectively measure both speed value and direction of the wind between canopies (**Figure 12B**). The wind speed in X direction was the core (about from -2.5 m·s^-1^ to -3.5 m·s^-1^), while that in the other two directions was approximately equal in absolute value (between 1.0 m·s^-1^ and 1.5 m·s^-1^). Meanwhile, the wind direction could be known based on the sign of the values. Thus, the wind in the X and Y direction of the SCS was upward along the tube, while that in the Z direction of the SCS was downward.

(3) The wind field of the fan was not strictly parallel to the ground (**Figure 12C**). Wind vectors were at an angle with the X, Y and Z direction, which might be caused by canopy obstruction and mechanical accuracy of the fan (such as assembly accuracy and levelling). At the end of data acquisition (after about the 38^th^ data number), the vector pointed in the opposite direction due to the elasticity of the cantilever pieces. Thus, the system could reflect the whole process of the pieces from compression (wind blowing) to recovery (stopping blowing).

(4) The resultant wind speed was about 3 m·s^-1^, within the range from about 2.25 m·s^-1^ to 3.75 m·s^-1^(**Figure 12D**). The fluctuation might result from the elasticity of the cantilever pieces and the cycle of fan rotation. Combined with **Figure 12C**, it is identified that the wind between canopies was relatively stable when the fan was working.

Based on the results, if the fan is used to conduct air-assisted spray parallel to the ground, it should get attention to the possibility of excessive droplet depositions and drifts at the upper regions of target tree canopies.

### 3.3 Discussions

In this paper, a detection method for multiple parameters of wind-field was developed. The idea was inspired by the practice (Yang et al, 2022a) and the literature review (Yang et al, 2019c) by the authors. According to calibrations, the average measurement error of wind-speed values was small (less than 5%), and wind direction could also be synchronously detected. In addition, the real application for measuring the wind between canopies generated by the fan, SFG4-2R, justified that the method could not only directly obtain the speed and direction of wind fields but also help analyze wind-field distributions of specific air-assisted devices. This highlights the practical application value of the proposed method and system. Thus, the method was able to effectively deal with the issue of synchronous three-dimensional measurement of wind speed and direction.

Moreover, it is a common difficulty for not only agricultural but non-agricultural fields to achieve simultaneous detection of wind speed and direction in three dimensions. Combined with a novel structure for wind sensing and its calculation models on the basis of the relations between strains and wind speed (from strains to wind thrust to wind pressure to wind speed), stereo wind measurement could be achieved based on the vectors calculated by strains, which means that the method is general and adequate for any wind-field measurement within the sensing range, not only restricted to be used in air-assisted spray conditions. For instance, for the ventilation design of nursery pig houses, the conventional approach for Computational Fluid Dynamics (CFD) verification was just by evaluating wind speed errors (Fang et al, 2022). It might be better to use the proposed method to obtain wind speed and direction at the same time to achieve multi-parameter verifications. In terms of mining, sufficient wind speed can dilute harmful gases to ensure the safety of operators, so it is important to measure accurate wind speed in downhole situations. Compared with the previous contribution (Xue et al, 2022), this proposed method could give 3D results while ensuring accuracy, and then help to predict the potential spatial distribution of gas. If the method is applied to measure the wind fields with a speed of more than 15 m/s, the materials of cantilever pieces and the calibration range should be changed.

Furthermore, it should be pointed out that an open wind tunnel was exploited for calibration, which was the ONLY resource that can be found during the epidemic, even though a closed one may be more suitable due to low turbulence and uniformed wind. Nonetheless, it was not caused by the limitation of the method itself and can be addressed. For future study, a closed wind tunnel and higher precision anemometers will be taken as standard devices for conducting much more precise calibrations.

In addition, different cantilever piece materials may form different vibration periods because of elasticity, which may influence data processing approaches. In the follow-up study, the effect of cantilever piece materials can be further analyzed. However, irrespective of any materials, the calculation of wind speed and wind direction (the equations from (8) to (14)) will not change. Only the variable values will differ, while the developed method is a general detection technique. Moreover, the impact of the system size could be further analyzed and adjusted based on measurement demand, while this paper mainly focuses on the feasibility and reliability of this new method. Therefore, these two issues were not examined in this paper.

## 4 Conclusions

This paper proposes a novel synchronous detection method with a regular triangular pyramid shape supported by cantilevers to deal with the difficulty of multi-parameter and multi-dimensional measurement of wind fields. The wind vector principle was utilized to develop the calculation models of values and directions of wind fields, which was related to the relationship of ‘strains-force-wind pressure-wind velocity’ and that of space operation of vectors, and tests were conducted. The conclusions are:

(1) Thermal anemometers (Testo-405i) and an open wind tunnel were used for calibration. Results showed that the minimum relative error of wind-speed value measurement was about 0.06%, while the maximum was about 10%. The average relative error of all the directions was less than 5%. It could be illustrated that the proposed method had a good measurement accuracy.

(2) The measurement of the wind among artificial tree canopies demonstrated that the proposed method could effectively measure both speed value and direction of the wind among canopies. Wind vectors could be clearly shown. Moreover, the possibility of bias-to-upper-part depositions and drifts of the fan, SFG4-2R, should be noticed according to the results using the method.

The results highlighted the value of practical application of this approach and showed a technical system solution for evaluating wind-field characteristics of air-assisted sprayers based on three-dimensional simultaneous measurement.

## Data availability statement

The original contributions presented in the study are included in the article/supplementary material. Further inquiries can be directed to the corresponding author.

## Author contributions

SY: Design, Methodology, Experiments, Data Processing and Manuscript Writing. WL: Design, Coding and Experiments. XL: Design and Discussions. ZW: Scheme and Discussions. YZ: Supervision and Funding. YT: Supervision and Funding. HF: Experiments. All authors contributed to the article and approved the submitted version.

## Acknowledgments

This study was funded by the National Natural Science Foundation of China (NSFC, 32171901) and the Research Innovation Fund for Graduate Students of China Agricultural University (2020XYZC38A). The wind tunnel used in the paper was supported by College of Water Resources and Civil Engineering, China Agricultural University. NI 9237 and compact DAQ 9135 used in the paper were supported by the team of Professor Zhenghe SONG and Bin XIE, College of Engineering, China Agricultural University. Meanwhile, thanks to Doctor Changkai WEN for specific details of the devices.

## Conflict of interest

The authors declare that the research was conducted in the absence of any commercial or financial relationships that could be construed as a potential conflict of interest.

## Publisher’s note

All claims expressed in this article are solely those of the authors and do not necessarily represent those of their affiliated organizations, or those of the publisher, the editors and the reviewers. Any product that may be evaluated in this article, or claim that may be made by its manufacturer, is not guaranteed or endorsed by the publisher.
